# A Nomogram for the Determination of the Necessity of Concurrent Chemotherapy in Patients With Stage II–IVa Nasopharyngeal Carcinoma

**DOI:** 10.3389/fonc.2021.640077

**Published:** 2021-09-06

**Authors:** Kaixuan Yang, Qian Zhang, Mengxi Zhang, Wenji Xie, Mei Li, Lei Zeng, Qiang Wang, Jianling Zhao, Yiping Li, Guangjun Li

**Affiliations:** ^1^Department of Gynecology and Obstetrics, Key Laboratory of Birth Defects and Related Diseases of Women and Children, Ministry of Education, West China Second University Hospital, Sichuan University, Chengdu, China; ^2^Department of Radiation Oncology, Cancer Center, West China Hospital, Sichuan University, Chengdu, China; ^3^Department of Radiation Oncology, Hunan Cancer Hospital & The Affiliated Cancer Hospital of Xiangya School of Medicine, Central South University, Changsha, China

**Keywords:** nasopharyngeal carcinoma, intensity-modulated radiotherapy, concurrent chemotherapy, nomogram, chemoradiotherapy

## Abstract

**Background:**

The efficiency of concurrent chemotherapy (CC) remains controversial for stage II–IVa nasopharyngeal carcinoma (NPC) patients treated with induction chemotherapy (IC) followed by intensity-modulated radiotherapy (IMRT). Therefore, we aimed to propose a nomogram to identify patients who would benefit from CC.

**Methods:**

A total of 434 NPC patients (stage II–IVa) treated with IC followed by IMRT between January 2010 and December 2015 were included. There were 808 dosimetric parameters extracted by the in-house script for each patient. A dosimetric signature was developed with the least absolute shrinkage and selection operator algorithm. A nomogram was built by incorporating clinical factors and dosimetric signature using Cox regression to predict recurrence-free survival (RFS). The C-index was used to evaluate the performance of the nomogram. The patients were stratified into low- and high-risk recurrence according to the optimal cutoff of risk score.

**Results:**

The nomogram incorporating age, TNM stage, and dosimetric signature yielded a C-index of 0.719 (95% confidence interval, 0.658–0.78). In the low-risk group, CC was associated with a 9.4% increase of 5-year locoregional RFS and an 8.8% increase of 5-year overall survival (OS), whereas it was not significantly associated with an improvement of locoregional RFS (LRFS) and OS in the high-risk group. However, in the high-risk group, patients could benefit from adjuvant chemotherapy (AC) by improving 33.6% of the 5-year LRFS.

**Conclusions:**

The nomogram performed an individualized risk quantification of RFS in patients with stage II–IVa NPC treated with IC followed by IMRT. Patients with low risk could benefit from CC, whereas patients with high risk may require additional AC.

## Introduction

The cases of newly diagnosed nasopharyngeal carcinoma (NPC) reached about 129,000 in 2018 (1). More than 70% of NPC patients are in the east and southeast Asia. NPC is relatively sensitive to radiotherapy; radiotherapy is the mainstay treatment modality for non­metastatic disease. As for stage II–IVa NPC, concurrent chemotherapy (CC) combined with radiotherapy is used to enhance radiosensitivity and local control. In the two-dimensional (2D) radiotherapy era, concurrent chemoradiotherapy (CCRT) is superior to radiotherapy in patients with stage III–IVa disease for progression-free survival (PFS) and overall survival (OS) (2). Stage II disease could also benefit from CCRT in terms of PFS and OS compared with radiotherapy alone in the 5- and 10-year follow-up (3, 4). Therefore, CCRT is widely accepted for treating stage II–IVa NPC (5).

Over time, radiotherapy modality has progressed from conventional 2D radiotherapy to 3D conformal radiotherapy and then to intensity-modulated radiotherapy (IMRT) for NPC. Recently, evidence from retrospective studies has shown that induction chemotherapy (IC) plus IMRT was superior to IC plus CCRT, with comparable survival outcomes and fewer acute toxic effects in treating stage II–IVa NPC (6). Notably, CCRT has a potential risk to increase the incidence of grade 3/4 acute toxicities (2, 6) and radiotherapy time, which adversely affect the survival of NPC (7). Therefore, it is critical to determine whether patients treated with IC followed by IMRT can benefit from CC. This can greatly enhance clinical decision-making.

IMRT is an advanced type of radiation therapy to conform the radiation dose to the tumor and to avoid the exposure of organs at risk (OARs) to enhance the therapeutic ratio. However, it is still challenging because of the anatomical proximity of gross tumor to OARs (8). Therefore, dosimetric inadequacy of target volumes that resulted from prescription constraints on critical OARs has been one of the most crucial independent prognosticators of survival outcomes of NPC (8–10). Dosimetric factors are commonly used to quantify the prescription isodose line coverage to the corresponding target volume and utilized to evaluate the plan quality among various radiotherapy approaches (11). It may improve patient care, too, by incorporating big data of dosimetric factors into clinical practice based on dose–volume histogram metrics of treatment plan (12, 13). Recently, an international guideline in radiotherapy planning for NPC highlights dose criteria (8). In addition, dosimetric factors are regarded as score criteria for radiotherapy plan comparison in the International Radiotherapy Plan Competition (https://radiationknowledge.org/). Therefore, precision medicine needs to quantify clinical treatment plans based on dosimetric factors.

In this study, we aimed at developing a prognostic nomogram based on IMRT dosimetric signature as a risk quantification model and further identifying patient subsets who can benefit from CC in patients with stage II–IVa NPC treated with IC followed by IMRT.

## Materials and Methods

### Study Workflow

**Figure 1:**
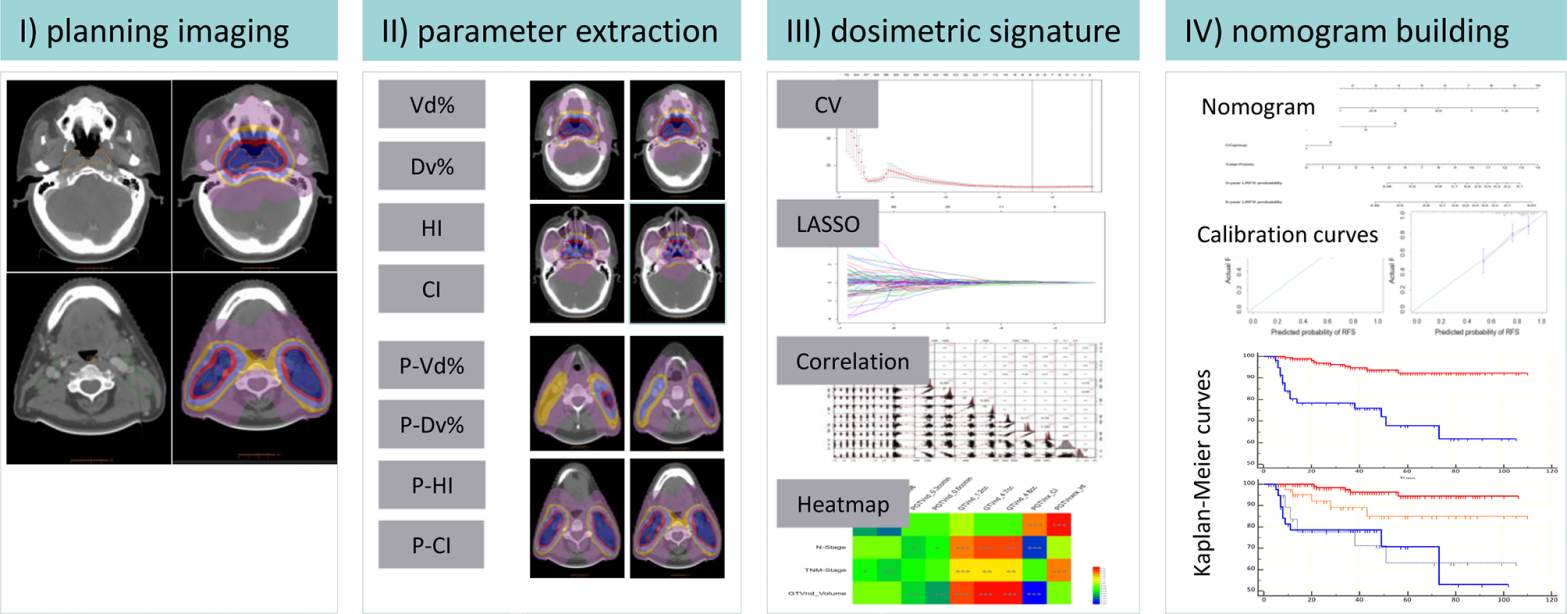
Schematic diagram of the proposed nomogram. CI, conformal index; HI, homogeneity index; Vd%, the volume exposed in d Gy; Dv%, the minimal dose delivered into v% of the volume; P-, the planning target volume; CV, cross-validation; LASSO, least absolute shrinkage and selection operator; GTVnx, nasopharynx gross tumor volume; GTVnd, lymph node gross tumor volume *P ≤ 0.05, **P ≤ 0.01, ***P ≤ 0.001.

**Figure 1** shows the workflow of this study, including (1) radiotherapy planning data restored, (2) dosimetric parameter extraction by in-house script, (3) dosimetric signature building, and (4) nomogram development and risk stratification for concurrent chemotherapy.

### Patients

This study was approved by our institutional review board, and the informed consent was waived because of the retrospective nature of this study. A dataset of 523 patients with stage II–IVa NPC (AJCC/UICC 8th edition) was reviewed from January 2010 to December 2015. The inclusion criteria were as follows: (1) patients with biopsy-confirmed primary NPC, (2) patients received cisplatin-based IC and radical IMRT, (3) patients received no previous treatments due to other cancers, and (4) patients with restored radiotherapy planning. The exclusion criteria were as follows: (1) failed to restore the radiotherapy planning (*n* = 14), (2) no available radiotherapy data in mosaiq system (*n* = 3), and (3) radiotherapy planning could not be extracted by the in-house script (*n* = 16). **Supplementary Figure S1** presented the patient inclusion flowchart. Finally, 434 patients were included, of which 247 patients (IC ± CC cohort) were treated with IC + CCRT or IC + IMRT and 187 (IC ± CC + AC cohort) were treated with IC + CCRT + AC or IC + IMRT + AC.

The characteristics of the patients were collected from the hospital information systems. The chemotherapy and radiotherapy regimens are described in **Supplementary S1**. The primary endpoints included RFS, locoregional RFS (LRFS), distant metastasis-free survival (DMFS), and OS. RFS was defined as the time from treatment until the diagnosis of a recurrent disease. LRFS was defined as the time from treatment to local or regional recurrence. DMFS was defined as the time from treatment to data of distant recurrence. OS was defined as the date from treatment to death from any cause.

### Dosimetric Parameter Extraction and Dosimetric Signature Building

The radiotherapy planning data were restored from the mosaiq system (Elekta Medical Systems, Sunnyvale, CA, USA) and were transferred to the treatment planning system Raystation. A total of 808 dosimetric parameters were calculated from the target volume for each patient through our in-house script (14), including D1-100, D1 cc-5 cc, D1min cc-5min cc. Dv% was described as the minimal dose delivered into v% of the target volume. Dv cc and Dvmin cc are the maximal and minimal dose exposed in the absolute target volume v cc. Gross tumor volume (GTV) contains the gross tumor volume of the nasopharynx (GTVnx) and gross tumor volume of regional lymph node (GTVnd). The planning GTVnx/nd (PGTVnx/nd) was obtained by expanding the corresponding GTVnx/nd with an expanded margin of 3 mm. GTVnx, GTVnd, PGTVnx, and PGTVnd were the target volumes. The least absolute shrinkage and selection operator (LASSO) algorithm was applied to select the dosimetric parameters that associated with RFS among the above-mentioned 808 features. The selected parameters were weighted by their respective coefficients and were calculated as a linear combination to construct the planning score for each patient.

### Development and Validation of Nomogram

Univariate and multivariate Cox proportional hazards regression model was applied to achieve the candidate variables. A nomogram predicting RFS was constructed based on the candidate variables of 247 patients from the IC ± CC cohort and was internally validated by performing 1,000 bootstrap resamples. The concordance index (C index) was utilized to evaluate the predictive performance of the nomogram. In addition, a calibration curve was plotted to compare the actual probabilities against the prediction probabilities of the nomogram.

### Classification of Patients Into Low- or High-Risk Subgroup

We computed the nomogram risk score for each patient by linear predictor function “predict” in R, which was applied for prediction based on the results of Cox model fitting functions (14). The cutoff value of the nomogram risk score was calculated by the X-tile software (version 3.6.1; Yale University, New Haven, CT, USA), which produced the largest *χ*² value in the Mantel–Cox test (15). The 247 patients were divided into low- or high-risk group according to the optimal cutoff risk score, and the same classification criterion was performed on the 187 patients.

### Statistical Analysis

All statistical analyses were performed on R software (version 3.5.2), MedCalc (version 15.6), and SPSS (version 22.0). The *t*-test was used for comparison between continuous variables, and chi-square test was used for comparison between categorical variables. All dosimetric and clinical data were normalized using log-transform before analysis. Kaplan–Meier curves were plotted by MedCalc and compared by log-rank test. R packages were used as follows: “glment” for LASSO–Cox regression, “Rms” for nomogram and calibration curves, “Hmisc” for correlation matrix, and “HemI” (Heatmap Illustrator, version 1.0) for heat map. “ipw” (**Supplementary S2**) was applied for estimating the inverse probability weights to control confounding (16).

## Results

### Patient Characteristics

Patient characteristics and cycles of IC were compared between the CC and non-CC cohorts and between the AC and non-AC cohorts (**Table 1**). No significant differences were observed between the two cohorts besides age (*p* = 0.001) and hemoglobin (*p* = 0.04) in the IC ± CC cohort. Forty (9.2%) patients were at stage II. After a median follow-up of 50 months (range, 2–113 months), 44 patients experienced locoregional recurrence, 64 patients experienced distant recurrence, and 99 patients died of NPC. For the whole dataset, the 5-year LRFS, DMFS, and OS rates were 85.5, 82.4, and 83.8%, respectively.

**Table 1:** Characteristics of all patients (*n* = 434) with nasopharyngeal carcinoma.

	IC ± CC cohort			IC ± CC + AC cohort		
	Non-CC	CC	*P*-value	Non-CC	CC	*P*-value
	(*n* = 67)	(*n* = 180)		(*n* = 59)	(*n* = 128)	
Age (years)	53.3 ± 11.3	46.6 ± 10.3	0.001	48.5 ± 10.7	44.5 ± 9.9	0.344
Sex			0.274			0.102
Male	46 (69)	136 (76)		32 (54)	53 (41)	
Female	21 (31)	44 (24)		27 (46)	75 (59)	
BMI (kg/m^2)^	22.3 ± 3	22.6 ± 3.1	0.545	22.5 ± 2.7	22.5 ± 3.9	0.678
Family history			0.741			0.512
No	57 (85)	150 (83)		46 (78)	105 (82)	
Yes	10 (15)	30 (17)		13 (22)	23 (18)	
Cigarette smoking			0.619			0.635
No	30 (45)	87 (48)		34 (58)	69 (54)	
Yes	37 (55)	93 (52)		25 (42)	59 (46)	
Alcohol consumption			0.906			0.997
No	50 (75)	133 (74)		47 (80)	102 (80)	
Yes	17 (25)	47 (26)		12 (20)	26 (20)	
Hb (g/L)	139.0 ± 16.4	143.2 ± 14.4	0.040	137.9 ± 16	136.6 ± 22.9	0.910
PLT (×10^9^/L)	184 ± 54.3	192.2 ± 66.9	0.942	191 ± 67.9	204.3 ± 57.7	0.504
Neutrophil count (×10^9^/L	4.1 ± 1.6	6.9 ± 0.8	0.386	3.7 ± 1.4	3.8 ± 1.3	0.533
Lymphocyte count (×10^9^/L	1.6 ± 0.6	1.7 ± 0.6	0.321	1.5 ± 0.7	1.7 ± 0.7	0.333
NLR	2.8 ± 1.6	5.8 ± 1.3	0.477	2.7 ± 0.4	2.5 ± 1.3	0.457
LDH (IU/L)	175.4 ± 57.9	179.5 ± 75	0.941	170.1 ± 44	171.8 ± 48.5	0.591
AJCC/UICC 8th TNM stage			0.730			0.672
II	6 (10)	20 (11)		4 (7)	10 (8)	
III	25 (37)	73 (41)		26 (44)	64 (50)	
IVa	36 (53)	87 (48)		29 (49)	54 (42)	
T stage			0.134			0.568
T1	12 (18)	21 (12)		7 (12)	19 (15)	
T2	4 (6)	25 (14)		8 (14)	19 (15)	
T3	23 (34)	74 (41)		34 (57)	64 (50)	
T4	28 (42)	60 (33)		10 (17)	26 (20)	
N stage			0.219			0.600
N0	2 (3)	1 (0)		0 (0)	1 (0)	
N1	11 (16)	46 (26)		7 (12)	20 (16)	
N2	39 (58)	96 (53)		30 (51)	70 (55)	
N3	15 (22)	37 (21)		22 (36)	37 (29)	
GTVnx (cc)	45.8 (25.8-63.9)	49.9 (33.1-74.7)	0.240	31.9 (21.4-55.5)	37.5 (25.7-63.2)	0.210
GTVnd (cc)	11.1 (5.8-22.2)	12.8 (7.1-27.9)	0.254	10.6 (6.9-20.7)	14.2 (8.2-30.7)	0.099
WHO histological type			0.246			0.419
Keratinizing SqCC	0 (0)	0 (0)		0 (0)	1 (1)	
Non-keratinizing, differentiated	60 (90)	166 (92)		54 (92)	106 (83)	
Non-keratinizing, undifferentiated	4 (6)	12 (7)		3 (5)	15 (12)	
Unspecified	3 (4)	2 (1)		2 (3)	6 (5)	
IC cycles			0.33			0.292
≤2 cycles	35 (52)	92 (51)		40 (69)	78 (61)	
>2 cycles	32 (48)	88 (49)		18 (31)	50 (39)	

Data are shown as mean (standard deviation), median (interquartile ranges), or number (percentage).

BMI, body mass index; Hb, hemoglobin; PLT, platelets; NLR, neutrophil lymphocyte ratio; LDH, lactate dehydrogenase; GTVnx, nasopharynx gross tumor volume; GTVnd, lymph node gross tumor volume; cc, cubic centimeter; CC, concurrent chemotherapy; IC, induction chemotherapy; AC, adjuvant chemotherapy.

### Dosimetric Signature Construction

Of the 808 dosimetric parameters, 404 metrics were extracted from the GTV, and the remaining 404 were extracted from the planning gross tumor volume (PGTV). After LASSO, three GTV parameters (GTVnd_1.2cc, GTVnd_4.7cc, and GTVnd_4.8cc) and six PGTV parameters (PGTVnx_D98, PGTVnx_D99, PGTVnd_0.3ccmin, PGTVnd_0.6ccmin, PGTVnx_CI, and PGTVnx_HI) associated with RFS were applied to construct the dosimetric signature. The dosimetric signature equation was as follows:

Dosimetric signature = -0.004 * PGTVnx_D98 -0.093 * PGTVnx_D99 - 0.017 * PGTVnd_0.3ccmin - 0.087 * PGTVnd_0.6ccmin + 0.053 * GTVnd_1.2cc + 0.001 * GTVnd_4.7cc + 0.145 * GTVnd_4.8cc - 0.187 * PGTVnx_CI + 0.111 * PGTVnx_HI

### Association Between Dosimetric Parameters and Tumor-Related Data

**Figure 2:**
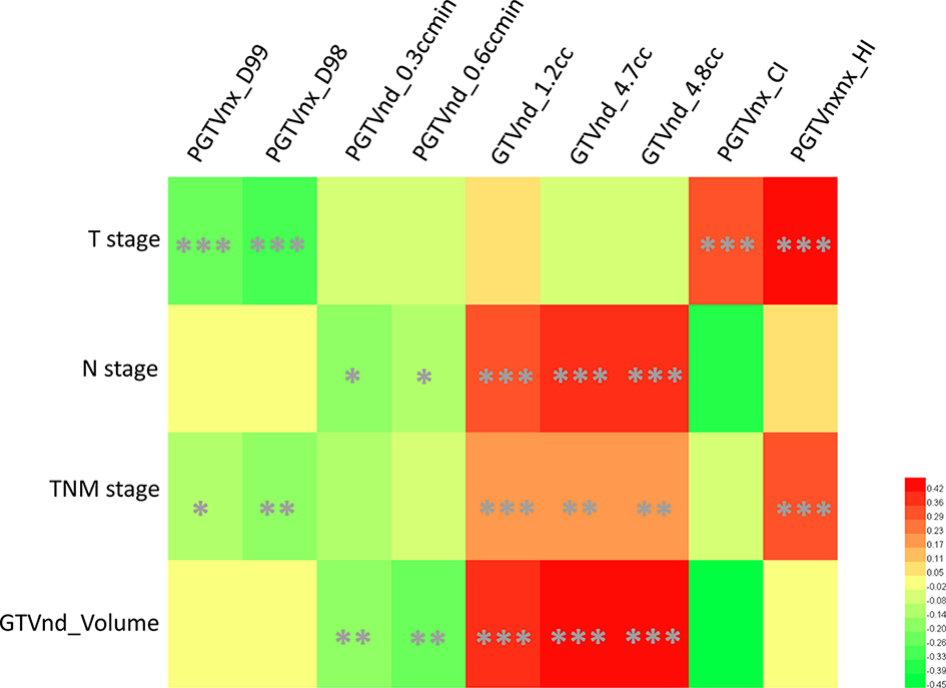
A heat map of associations between selected dosimetric parameters and tumor-related data. *P ≤ 0.05, **P ≤ 0.01, ***P ≤ 0.001.

In the IC ± CC cohort, the univariate Cox analysis identified T stage, N stage, TNM stage, and volume of GTVnd as tumor-related risk factors of RFS (**Table 2**). We then plotted the correlation matrix and heat map to illustrate the association between the nine dosimetric parameters and tumor-related data in **Supplementary Figure S2** and **Figure 2**, respectively. The results showed that T stage was significantly associated with four dosimetric parameters, including PGTVnx_CI, PGTVnx_HI, PGTVnx_D98, and PGTVnx_D99 (all *p ≤*0.001). N stage and GTVnd_Volume were associated with the same five dosimetric parameters, including PGTVnd_0.3ccmin, GTVnd_4.8cc, PGTVnd_0.6ccmin, GTVnd_1.2cc, and GTVnd_4.7cc (all *P* ≤0.05). TNM stage was associated with six dosimetric parameters, including PGTVnx_D99, PGTVnx_D98, GTVnd_1.2cc, GTVnd_4.7cc, GTVnd_4.8cc, and PGTVnxnx_HI (all *P* ≤0.05).

**Table 2:** Identification of risk factors of recurrence-free survival by univariate and multivariate analysis in induction chemotherapy ± concurrent chemotherapy cohort.

	Univariate Cox regression		Multivariate Cox regression	
	HR (95% CI)	*P*-value	HR (95% CI)	*P*-value
Age (years)	1.013 (0.988–1.038)	0.316	1.018 (0.992–1.045)	0.110
Sex (male *vs.* female)	0.760 (0.409–1.411)	0.384	–	–
BMI (kg/m^2^)	0.988 (0.954–1.023)	0.494	–	–
Family history of cancer (yes *vs.* no)	0.703 (0.319–1.550)	0.382	–	–
Cigarette smoking (yes *vs.* no)	1.202 (0.712–2.029)	0.492	–	–
Alcohol consumption (yes *vs.* no)	0.784 (0.422–1.456)	0.441	–	–
Hb (g/L)	0.998 (0.985–1.012)	0.798	–	–
PLT (x10^9^/L)	0.999 (0.995–1.004)	0.806	–	–
Neutrophil count (× 10^9^/L)	1.001 (0.992–1.010)	0.826	–	–
Lymphocyte count (x 10^9^/L)	1.413 (0.899–2.221)	0.134	–	–
NLR	1.000 (0.992–1.009)	0.961	–	–
LDH (IU/L)	1.001 (0.998–1.004)	0.415	–	–
TNM stage	2.236 (1.356–3.686)	**0.002**	1.944 (1.161–3.255)	**0.012**
T stage	1.426 (1.030–1.973)	**0.032**	1.203 (0.824–1.757)	0.362
N stage	2.066 (1.358–3.145)	**0.001**	1.233 (0.716–2.123)	0.637
GTVnx (cc)	1.002 (0.994–1.010)	0.632	–	–
GTVnd (cc)	1.010 (1.006–1.015)	**<**0.001	1.004 (0.998–1.010)	0.246
WHO type	1.206 (0.930–1.564)	0.157	–	–
IC+CCRT *vs.* IC+IMRT	0.467 (0.276–0.792)	**0.005**	0.607 (0.352–1.049)	0.071
IC cycles	1.113 (0.753–1.647)	0.591	–	
Dosimetric signature	4.825 (2.680–8.688)	**<**0.001	4.278 (2.360–7.755)	**<0.001**

HR, hazard ratio; CI, confidence intervals; BMI, body mass index; Hb, hemoglobin; PLT, platelets; NLR, neutrophil lymphocyte ratio; LDH, lactate dehydrogenase; GTVnx, nasopharynx gross tumor volume; GTVnd, lymph node gross tumor volume; cc, cubic centimeter; IMRT, intensity-modulated radiotherapy.

Statistically significant values (*P ≤ 0.05) were formatted in bold.

### Development of the Prognostic Nomogram

**Figure 3:**
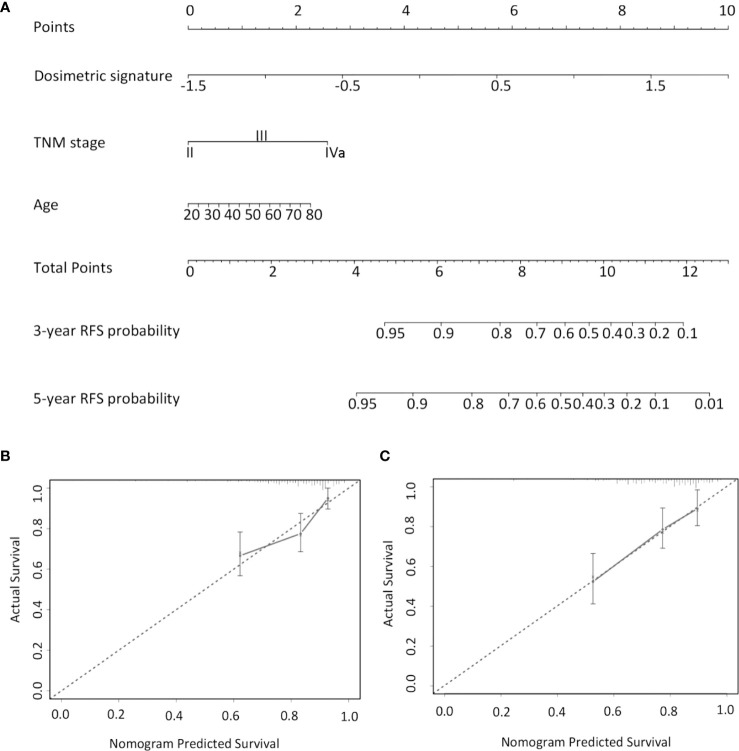
Nomogram for predicting 3- and 5-year RFS. **(A)** For each patient, the values of three variables (age, TNM stage, and dosimetric signature) are represented as points by projecting them onto the upper-most line (point scale). Summing the three variables and projecting the total points value downward onto the bottom-most line can determine the probability of 3- and 5-year RFS. Calibration curves of the nomogram in predicting 3- **(B)** and 5-year RFS **(C)**. The X-axis indicates the predicted probabilities of RFS, while the y-axis shows the actual RFS. RFS, relapse-free survival.

A subsequent multivariate Cox analysis showed that dosimetric signature (HR, 4.278, 95% CI: 2.36–7.755) and TNM stage (HR, 1.944, 95% CI: 1.161–3.255) were two independent risk factors for RFS (**Table 2**). As age is a key consideration in the NPC treatment, a nomogram incorporating age, TNM stage, and dosimetric signature to predict the 3- and 5-year RFS was developed in the IC ± CC cohort (**Figure 3A**). The nomogram was internally validated by bootstrapping for 1,000 times. The unadjusted and bootstrapping-corrected C-index values of the nomogram in predicting RFS were 0.712 (95% CI: 0.647–0.777) and 0.719 (95% CI: 0.658–0.78), respectively. The calibration curves showed a good agreement between the predicted 3- and 5-year RFS probabilities by nomogram and actual 3- and 5-year RFS (**Figures 3B, C**).

### Chemotherapy in Patient Groups With Low and High Risk

**Figure 4:**
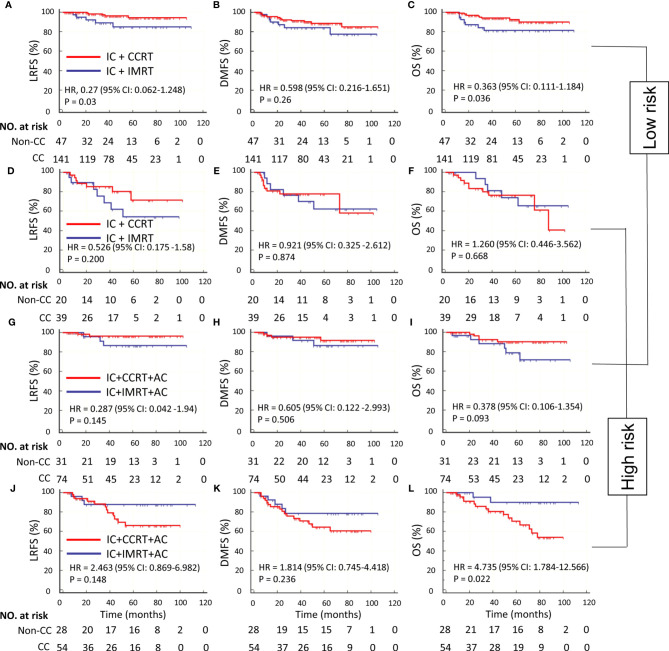
Kaplan–Meier survival curves of LRFS, DMFS, and OS in IC ± CC cohort with low risk **(A–C)**, IC ± CC cohort with high risk (D–F), IC ± CC + AC cohort with low risk **(G–I)**, and IC ± CC + AC cohort with high risk **(J–L)**. CC, concurrent chemotherapy; IC, induction chemotherapy; AC, adjuvant chemotherapy; IMRT, intensity-modulated radiotherapy; CCRT, concurrent chemoradiotherapy; LRFS, locoregional recurrence-free survival; DMFS, distant metastasis-free survival; OS, overall survival.

We performed a subset analysis to evaluate the survival benefit of CC in low- and high-risk groups. According to the optimal cutoff risk score of 0.60 identified by the X-tile plot, the patients were stratified into low-risk group (<0.60) and high-risk groups (≥0.60). The Kaplan–Meier curves showed significantly different LRFS, DMFS, and OS between the two groups (**Supplementary Figure S3**). In the low-risk group, IC + CCRT was superior to IC + IMRT in terms of LRFS (HR, 0.27; 95% CI: 0.062–1.248; *P* = 0.03) and OS (HR, 0.363; 95% CI: 0.111–1.184; *P* = 0.036) but not DMFS (HR, 0.598; 95% CI: 0.216–1.651; *P* = 0.26) (**Table 3** and **Figures 4A–C**). CC was associated with a 9.4% increase of 5-year LRFS and an 8.8% increase of 5-year OS (**Supplementary Figure S4**). IC + CCRT + AC was not superior to IC + IMRT + AC in terms of LRFS, DMFS, and OS (*P* = 0.145, 0.506, and 0.093, respectively) (**Table 3** and **Figures 4G–I**). In the high-risk group, IC + CCRT was not superior to IC + IMRT in terms of LRFS, DMFS, and OS (*P* = 0.2, 0.874, and 0.668, respectively); IC + CCRT + AC was not superior to IC + IMRT + AC in terms of OS (HR, 4.735; 95% CI: 1.784–12.566; *P* = 0.022) and LRFS and DMFS (*P* = 0.148 and 0.236, respectively) (**Table 3** and **Figures 4D–F, J–L**). The aforementioned results were confirmed by IPTW (data not shown) to control confounding.

**Table 3:** Effects of concurrent chemotherapy on LRFS, DMFS, and OS in different subgroups according to the risk score of the nomogram.

	CC	Non-CC	LRFS		DMFS		OS	
			*P*-value	HR (95% CI)	*P*-value	HR (95% CI)	*P*-value	HR (95% CI)
IC cohort (*n* = 247)								
Low risk	141	47	**0.030**	0.270 (0.062–1.248)	0.260	0.598 (0.216–1.651)	**0.036**	0.363 (0.111–1.184)
High risk	39	20	0.200	0.526 (0.175–1.580)	0.874	0.921 (0.325–2.612)	0.668	1.260 (0.446–3.562)
All patients	180	67	0.004	0.329 (0.130–0.832)	0.185	0.635 (0.303–1.333)	0.093	0.544 (0.243–1.220)
IC+AC cohort (*n* = 187)								
Low risk	74	31	0.145	0.287 (0.042–1.940)	0.506	0.605 (0.122–2.993)	0.093	0.378 (0.106–1.354)
High risk	54	28	0.148	2.463 (0.869–6.982)	0.236	1.814 (0.745–4.418)	**0.022**	4.735 (1.784–12.566)
All patients	128	59	0.757	1.163 (0.459–2.948)	0.590	1.252 (0.571–2.746)	0.544	1.287 (0.589–2.813)

CC, concurrent chemotherapy; IC, induction chemotherapy; AC, adjuvant chemotherapy; LRFS, locoregional recurrence-free survival; DMFS, distant metastasis-free survival; OS, overall survival.

Statistically significant values (*P ≤ 0.05) were formatted in bold.

We further explored the additional survival benefit of AC in both the low- and high-risk groups. In the low-risk group, IC + IMRT + AC was not superior to IC + IMRT in terms of LRFS, DMFS, and OS (*P* = 0.812, 0.436, and 0.851, respectively); IC + CCRT + AC was not superior to IC + CCRT in terms of LRFS, DMFS, and OS (*P* = 0.862, 0.316, and 0.771, respectively) (**Supplementary Table S1** and **Supplementary Figures S5A–C, G–I**). In the high-risk group, IC + IMRT + AC was superior to IC + IMRT in terms of LRFS (HR, 0.272; 95% CI 0.076–0.969; *P* = 0.042) but not DMFS and OS (*P* = 0.341 and 0.119, respectively) (**Supplementary Table S1** and **Supplementary Figurea S5D–F**), AC was associated with a 33.6% increase of 5-year LRFS (**Supplementary Figure S4**), and IC + CCRT + AC was not superior to IC + CCRT in terms of LRFS, DMFS, and OS (*P* = 0.757, 0.779, and 0.964, respectively) (**Supplementary Table S1** and **Supplementary Figures S5J–L**).

## Discussion

Based on dosimetric parameters derived from the IMRT plan in patients with stage II–IVa NPC treated with IC followed by IMRT, we constructed a novel nomogram to quantify the risk of RFS of individuals. Using the optimal cutoff risk score calculated by the model, we successfully categorized the patients into low- and high-risk subgroups with significantly different survival outcomes. Patients with a low risk could benefit from CC by improving the 5-year LRFS and OS, while patients with a high risk could choose IC + IMRT + AC to obtain an improved 5-year LRFS.

A total of 808 dosimetric factors were extracted and reduced to a panel of nine potential prognosticators by using a LASSO–Cox. LASSO is a robust regression technique for penalized analysis and variable selection from the mass data of dosimetric parameters (14, 17, 18). Cox regression analysis recognized the dosimetric signature, composed of nine factors, as an independent risk factor of RFS even after adjustment for age, TNM stage, and other confounders. Dosimetric signatures were essentially generated from radiotherapy plans based on contours that are a product of disease extent (TNM stage and/or disease volume). As potential confounders (19), disease extent should be incorporated into a regression analysis (**Table 2**). Unexpectedly, it was TNM stage, not disease volume, that associated with RFS. The reasons for this result can be explained by the inadequate tumor coverage (reflected by the dosimetric signature) that were mainly from TNM stage (adherence to adjacent critical structures) rather than disease volume in the IMRT era (8). It is clinically challenging to strike a balance between the risk of recurrence due to inadequate tumor coverage and radiation complications of the adjacent critical structures.

Considering that inadequate tumor coverage is mainly from the disease extent in the IMRT era, the association between dosimetric signature and tumor characteristics was investigated in this study. A higher T stage was related to higher conformal index and homogeneity index. With the primary tumor infiltrating deeply, the margins between gross tumor and nearby critical OARs become narrower and narrower. Because of the radiation safety principle “as low as reasonably practicable” for critical OARs in clinical practice (8), a steeper dose fall-off outside the target volume and a better conformal index are prone to be observed in advanced T stage patients (20, 21). As a compromise to the critical OARs, inhomogeneous target dose distribution is a strategy without an apparent dosimetric inadequacy of target volumes (22). However, inhomogeneous plans may be attributable to more cold spots (23), as the representative factors of cold spots, PGTVnx_D98 or PGTVnx_D99, seem to be ignored by PGTVnx_D95 in plan evaluation (24). As for N stage and GTVnd_Volume, we found that all the dose factors were associated with the two regardless of the volume and dose. It may be due to the progressive deformation of neck soft tissue structures from intrafraction motion (25). In addition, a recent study suggested that positive regional lymph nodes should be focused prior to primary tumor in NPC radiotherapy (14).

Our dosimetric signature does not contain D95 that is often used in the radiotherapy area. A plausible explanation for this might be that more attention on D95 in daily clinical practice limits its power. Definitive chemoradiotherapy is recommended by the 2021 National Comprehensive Cancer Network guidelines for stage II–IVa NPC patients. Because the quality of IMRT plans mainly relies on the experience of the dosimetrists (26), our dosimetric signature performed individualized risk quantification of IMRT plan for RFS. making it a surrogate for plan quality. It is therefore plausible that the chemotherapy benefit may be making up for poor radiation planning.

We found that CC was not a significant prognosticator of survival outcomes in the multivariate analysis. However, a subgroup analysis showed that CC can benefit patients with low risk instead of high risk in improving LRFS and OS. This finding is consistent with a gene expression-based signature study (27), a clinical experience-based report (28), and a dose–effect analysis (29). However, the results differed from other previous studies (30, 31), possibly because of the patients treated with 2D (31) or no comparison of radiotherapy alone in the IMRT subgroup analysis (30). We expected the results of an ongoing phase 3 clinical trial (NCT02633202) comparing CCRT with IMRT alone to establish the role of CC in the IMRT era.

Our study showed that AC can benefit high-risk patients treated with IC + IMRT in terms of LRFS. The modality of IC + IMRT + AC may be a promising strategy for the high-risk group in the IMRT era. A phase III trial proved that AC with intravenous cisplatin and 5-fluorouracil failed to improve failure-free survival and OS after CCRT but increased the toxic effects in NPC (32). However, efforts have been made to find the candidates for AC. A postradiotherapy plasma Epstein–Barr virus DNA screening failed to identify patients with positive treatment effects (33). Interestingly, a benefit of distant failure and overall survival was obtained in high-risk patients with modified oral chemotherapy agents (34, 35). Compared to platin-based chemotherapy, this kind of metronomic chemotherapy was associated with less toxicities, good compliance, and potential activation of immunity (36). A 2019 American Society of Clinical Oncology abstract (abstract no. 6046) reported that the total cumulative cisplatin dose (concurrent/induction/adjuvant) in multimodality therapy may be an independent prognosticator of LRFS and OS in locally advanced NPC. Therefore, the effect of CC was overemphasized, whereas the effect of AC was underestimated (34). Platin-based chemotherapy should be used post-IMRT instead of during IMRT in high-risk patients.

This study also has some limitations. Firstly, this was a retrospective study performed in a single center. Despite extensive adjustment, it is still possible that some amount of unmeasured confounders remains. Secondly, we did not consider the cumulative cisplatin dose because there was no consensus on the optimal cumulative cisplatin dose (29). Thirdly, EBV-DNA and its response after treatment is a major determinant of RFS and OS in patients with NPC (33, 37). However, the EBV-DNA in our datasets was measured at different time-points (*e*.*g*., post-IC, post-IMRT). A future update of the nomogram including the EBV data would strengthen the findings. Fourthly, setup error was not taken into consideration as a reason of limited daily CBCT data; a future study is needed to investigate the dosage inconsistency between treatment plan dose and the actual delivered dose which resulted from the error based on CBCT data. Finally, the clinical use of the nomogram can be tested if an external validation is executed.

## Conclusions

According to our knowledge, this is the first study to explore the role of CC in the viewpoint of IMRT-based dosimetric factors. Our findings revealed that the nomogram can effectively categorize patients with stage II–IVa NPC into subgroups with low and high risk of survival outcomes. A further analysis found that CC could improve LRR and OS for patients at low risk. Significantly, we identified that the high-risk patients could benefit from AC instead of CC. Prospective trials are needed to verify the positive treatment effect of CC and AC in low-r and high-risk patients, respectively. Our nomogram will allow clinicians to make more informed decisions for better patient care.

## Data Availability Statement

The original contributions presented in the study are included in the article/**Supplementary Material**. Further inquiries can be directed to the corresponding authors.

## Ethics Statement

The studies involving human participants were reviewed and approved by the Ethics Committee on Biomedical Research, West China Hospital of Sichuan University. The ethics committee waived the requirement of written informed consent for participation.

## Author Contributions

GL, KY: study design. KY, MZ, WX, ML, LZ, YL and QW: data collection. KY, JZ and QZ: data analysis. QZ, GL, and KY: data interpretation and writing.

## Funding

This study is funded by the National Natural Science Foundation of China (grant no. 81472807).

## Conflict of Interest

The authors declare that the research was conducted in the absence of any commercial or financial relationships that could be construed as a potential conflict of interest.

## Publisher’s Note

All claims expressed in this article are solely those of the authors and do not necessarily represent those of their affiliated organizations, or those of the publisher, the editors and the reviewers. Any product that may be evaluated in this article, or claim that may be made by its manufacturer, is not guaranteed or endorsed by the publisher.

## Supplementary Material

Supplementary Figure 1 The research recruitment pathway. IC, induction chemotherapy; AC, adjuvant chemotherapy; IMRT, intensity-modulated radiotherapy; CCRT, concurrent chemoradiotherapy.

Supplementary Figure 2 Visualization of the distribution and correlation coefficient from the selected dosimetric parameters and tumor-related data (diagonal coordinates are value of the predictors). **P* ≤ 0.05, ***P* ≤ 0.01, ****P* ≤ 0.001.

Supplementary Figure 3 Risk score group illustrated by Kaplan–Meier survival of LRFS, DMFS, and OS in the IC ± CC cohorts. The *P*-values were calculated using the log-rank test. HR, hazard ratio; LRFS, locoregional recurrence-free survival; DMFS, distant metastasis-free survival; OS, overall survival.

Supplementary Figure 4 Kaplan–Meier survival curves of LRFS, DMFS, and OS by four cohorts with low risk **(A–C)** and high risk **(D–F)**. CC, concurrent chemotherapy; IC, induction chemotherapy; AC, adjuvant chemotherapy; IMRT, intensity-modulated radiotherapy; CCRT, concurrent chemoradiotherapy; LRFS, locoregional recurrence-free survival; DMFS, distant metastasis-free survival; OS, overall survival.

Supplementary Figure 5 Kaplan–Meier survival curves of LRFS, DMFS, and OS in IC + IMRT ± AC cohort with low risk **(A–C)**, IC + IMRT ± AC cohort with high risk **(D–F)**, IC + CCRT ± AC cohort with low risk **(G–I)**, and IC + CCRT ± AC cohort with high risk **(J–L)**. CC, concurrent chemotherapy; IC, induction chemotherapy; AC, adjuvant chemotherapy; IMRT, intensity-modulated radiotherapy; CCRT, concurrent chemoradiotherapy; LRFS, locoregional recurrence-free survival; DMFS, distant metastasis-free survival; OS, overall survival.

Supplementary Table 1 Effects of adjuvant chemotherapy on LRFS, DMFS and OS in different subgroups according to risk sore of nomogram
