# Supplementary figures and images for: A Nomogram for the Determination of the Necessity of Concurrent Chemotherapy in Patients With Stage II–IVa Nasopharyngeal Carcinoma
Source: Front Oncol. 2021 Sep 6;11:640077. doi: 10.3389/fonc.2021.640077 (PMC8450530; doi:10.3389/fonc.2021.640077)

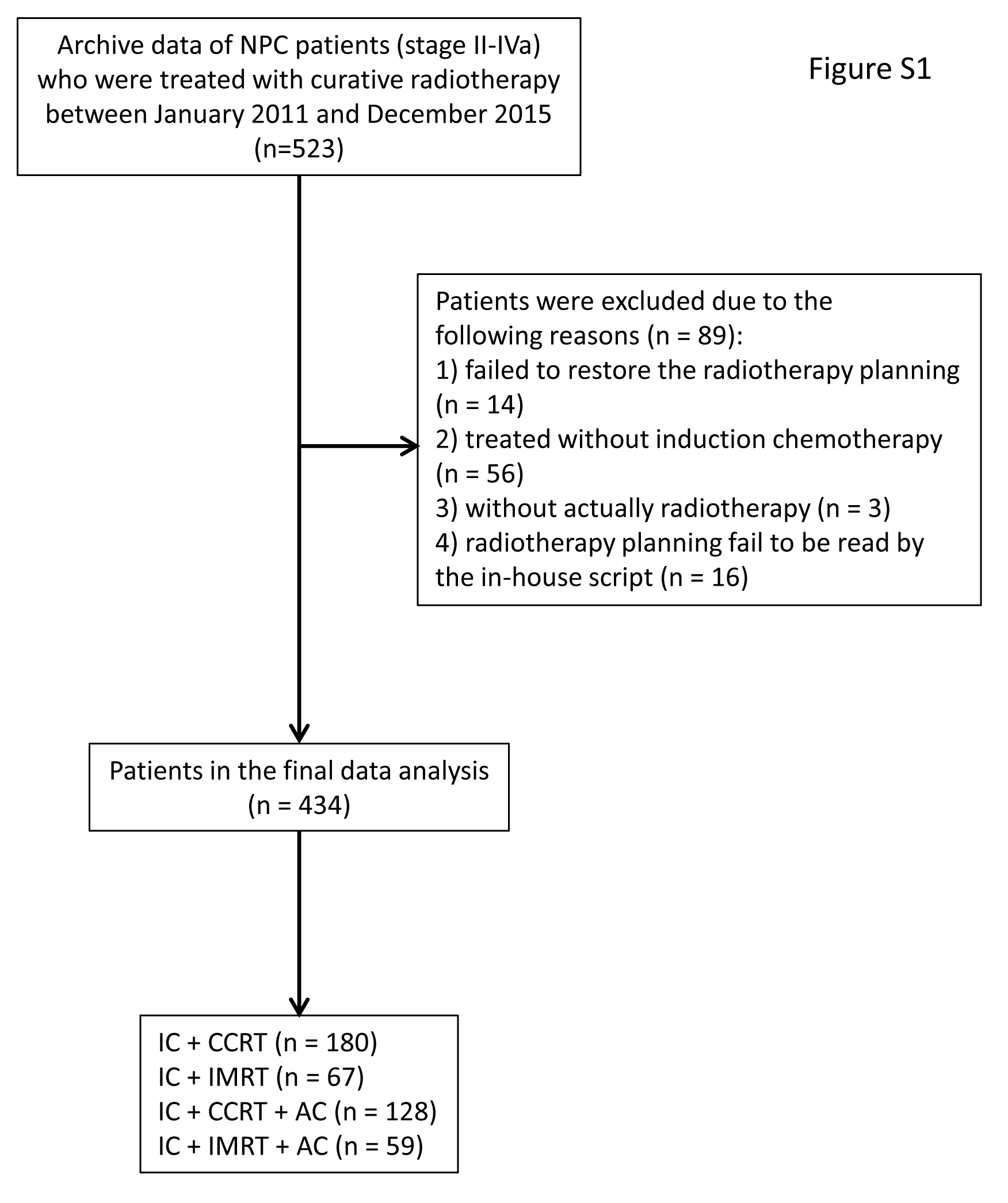

Supplement: Supplementary Figure 1 — The research recruitment pathway. IC, induction chemotherapy; AC, adjuvant chemotherapy; IMRT, intensity-modulated radiotherapy; CCRT, concurrent chemoradiotherapy. [file Image_1.tif]

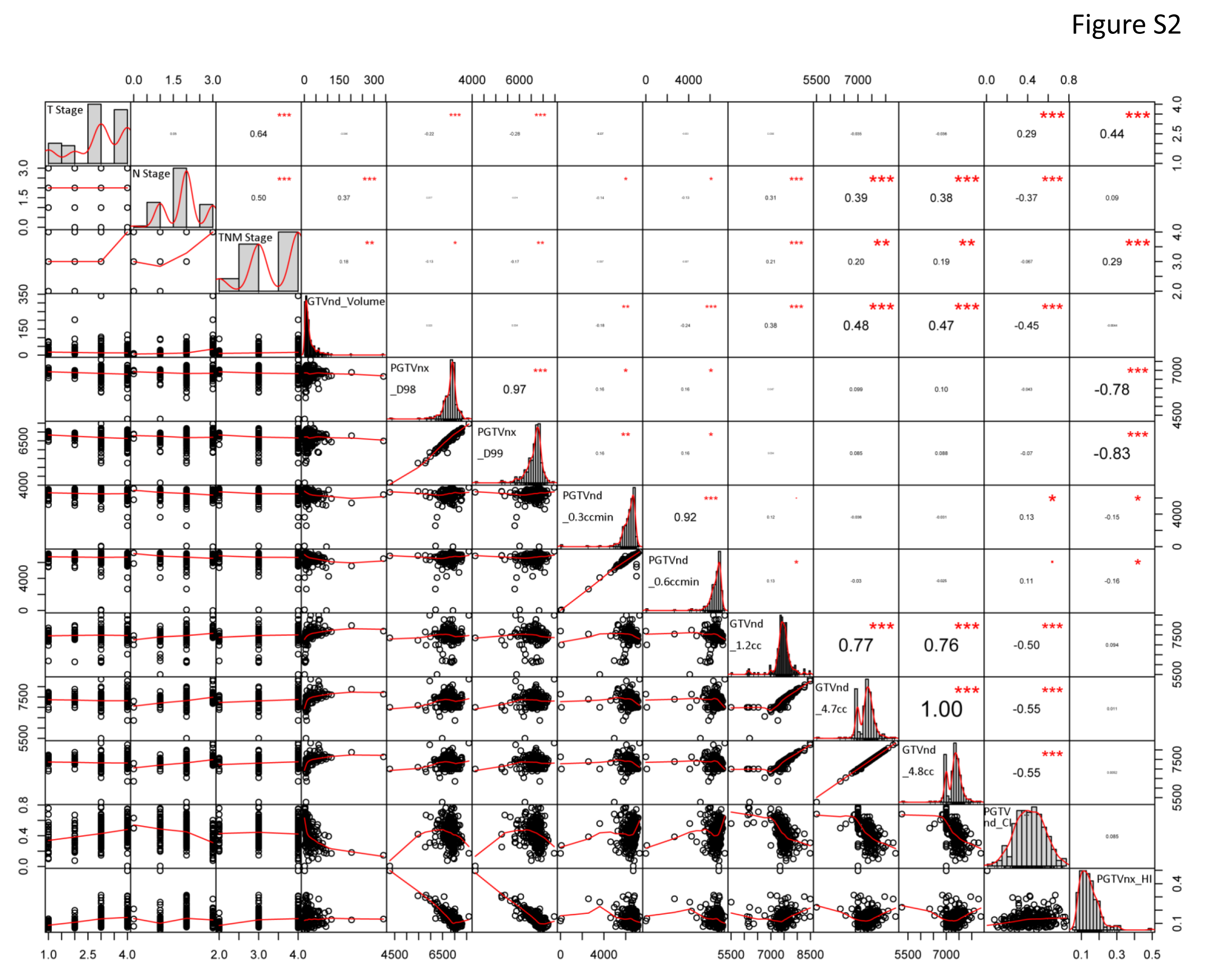

Supplement: Supplementary Figure 2 — Visualization of the distribution and correlation coefficient from the selected dosimetric parameters and tumor-related data (diagonal coordinates are value of the predictors). *P ≤ 0.05, **P ≤ 0.01, ***P ≤ 0.001. [file Image_2.tif]

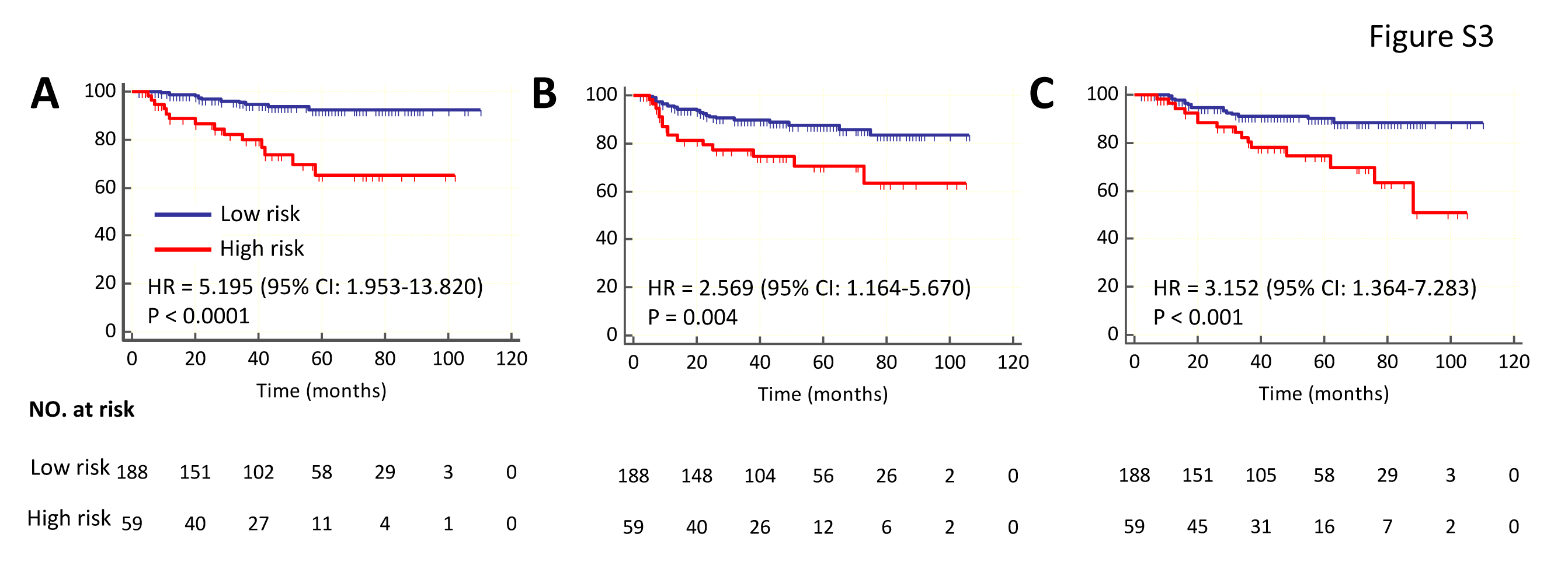

Supplement: Supplementary Figure 3 — Risk score group illustrated by Kaplan–Meier survival of LRFS, DMFS, and OS in the IC ± CC cohorts. The P-values were calculated using the log-rank test. HR, hazard ratio; LRFS, locoregional recurrence-free survival; DMFS, distant metastasis-free survival; OS, overall survival. [file Image_3.tif]

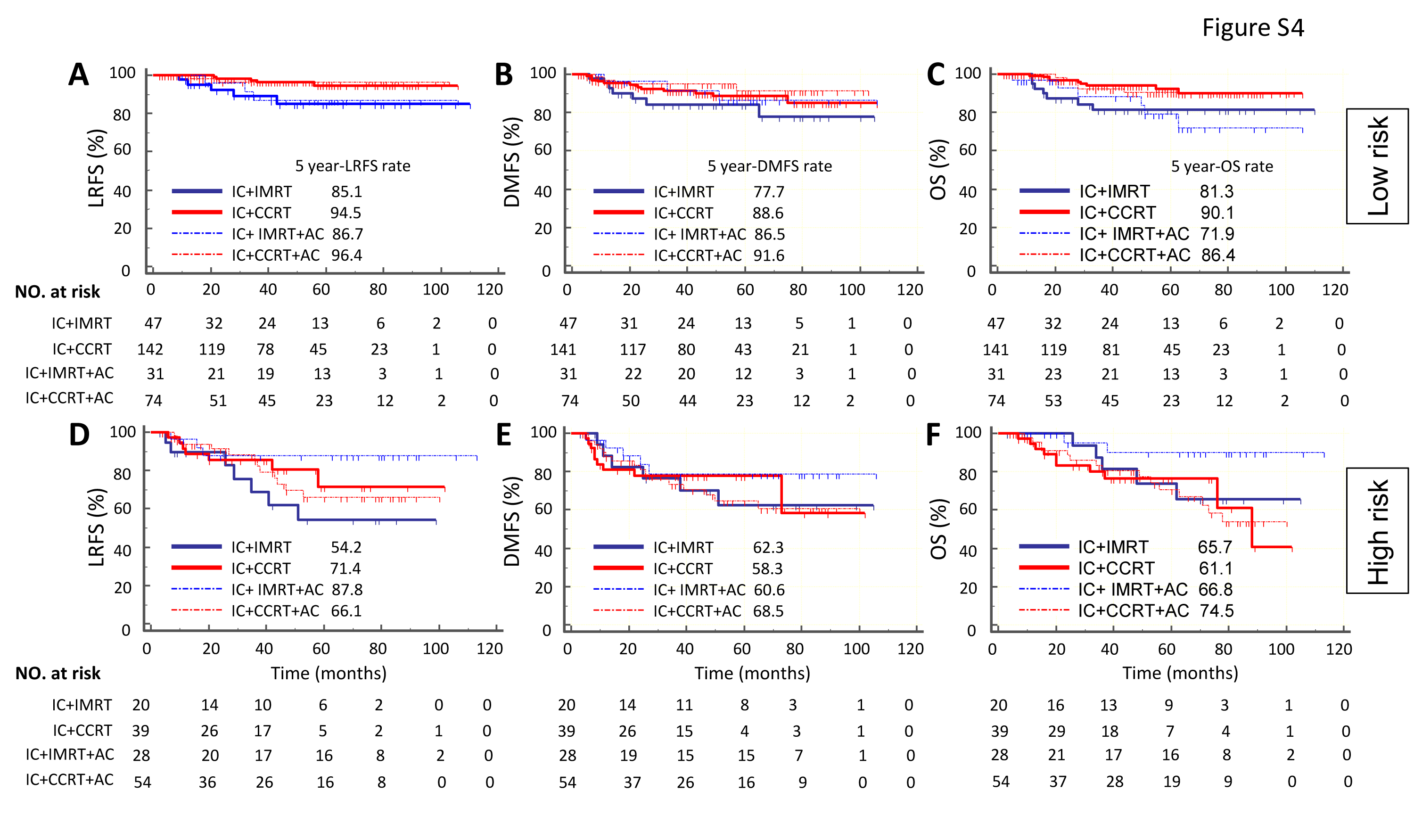

Supplement: Supplementary Figure 4 — Kaplan–Meier survival curves of LRFS, DMFS, and OS by four cohorts with low risk (A–C) and high risk (D–F). CC, concurrent chemotherapy; IC, induction chemotherapy; AC, adjuvant chemotherapy; IMRT, intensity-modulated radiotherapy; CCRT, concurrent chemoradiotherapy; LRFS, locoregional recurrence-free survival; DMFS, distant metastasis-free survival; OS, overall survival. [file Image_4.tif]

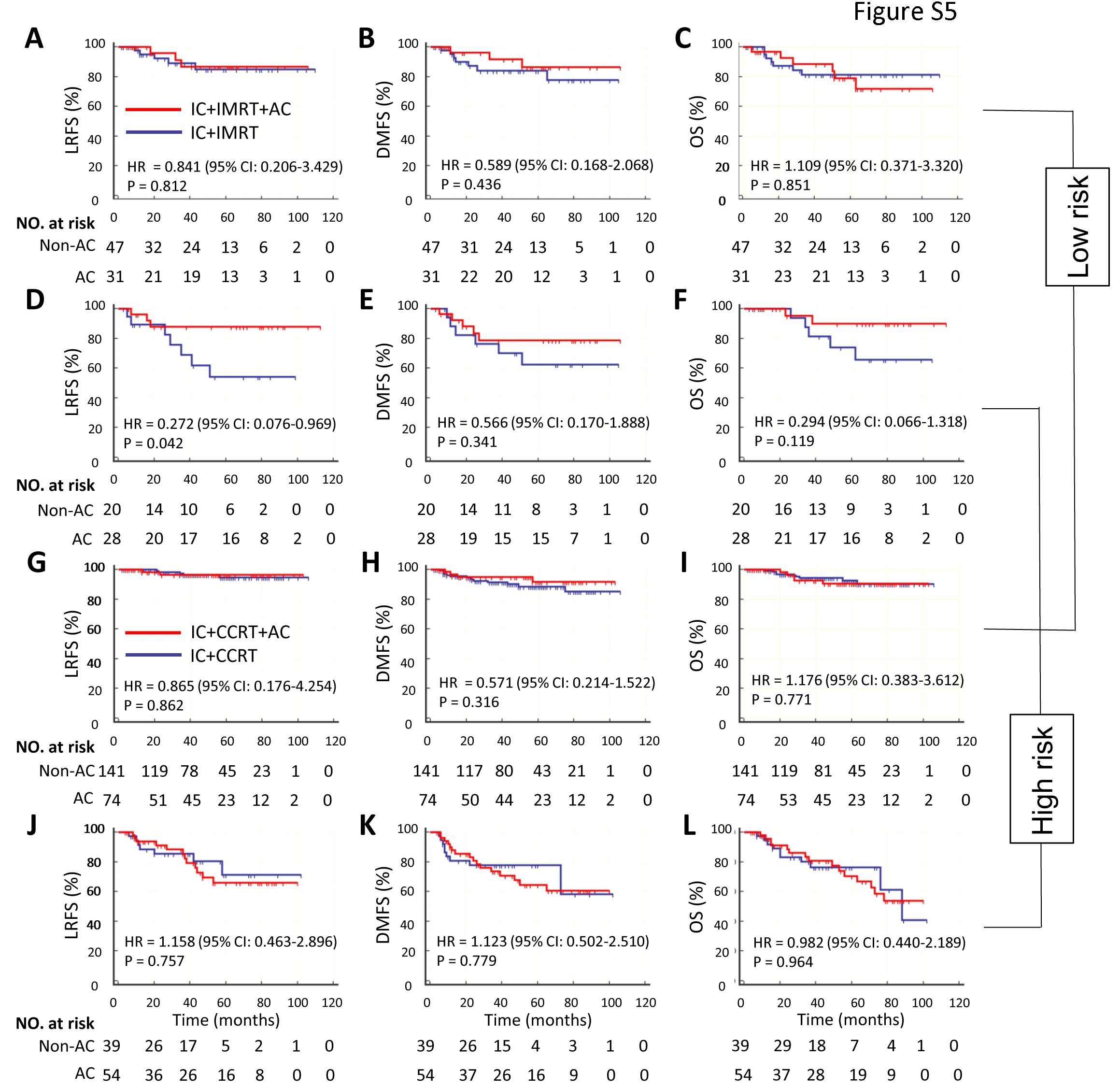

Supplement: Supplementary Figure 5 — Kaplan–Meier survival curves of LRFS, DMFS, and OS in IC + IMRT ± AC cohort with low risk (A–C), IC + IMRT ± AC cohort with high risk (D–F), IC + CCRT ± AC cohort with low risk (G–I), and IC + CCRT ± AC cohort with high risk (J–L). CC, concurrent chemotherapy; IC, induction chemotherapy; AC, adjuvant chemotherapy; IMRT, intensity-modulated radiotherapy; CCRT, concurrent chemoradiotherapy; LRFS, locoregional recurrence-free survival; DMFS, distant metastasis-free survival; OS, overall survival. [file Image_5.tif]
