# Supplementary material for: A Nomogram for the Determination of the Necessity of Concurrent Chemotherapy in Patients With Stage II–IVa Nasopharyngeal Carcinoma
Source: Front Oncol. 2021 Sep 6;11:640077. doi: 10.3389/fonc.2021.640077 (PMC8450530; doi:10.3389/fonc.2021.640077)
Supplement: Supplementary Table 1 — Effects of adjuvant chemotherapy on LRFS, DMFS and OS in different subgroups according to risk sore of nomogram [file Table_1.doc]

**Table S1. Effects of adjuvant chemotherapy on LRFS, DMFS and OS in different subgroups according to risk sore of nomogram**

|  | AC | Non- AC | LRFS | | DMFS | | OS | |
| --- | --- | --- | --- | --- | --- | --- | --- | --- |
|  |  |  | P value | HR (95% CI) | P value | HR (95% CI) | P value | HR (95% CI) |
| IC cohort (n=247) |  |  |  |  |  |  |  |  |
| Low-risk | 31 | 47 | 0.812 | 0.841 (0.206-3.429) | 0.436 | 0.589 (0.168-2.068) | 0.851 | 1.109 (0.371-3.320) |
| High-risk | 28 | 20 | **0.042** | 0.272 (0.076-0.969) | 0.341 | 0.566 (0.170-1.888) | 0.119 | 0.294 (0.066-1.318) |
| All patients | 59 | 67 | 0.161 | 0.504 (0.200-1.270) | 0.300 | 0.632 (0.268 -1.486) | 0.361 | 0.662 (0.276-1.590) |
| IC+CC cohort (n=187) |  |  |  |  |  |  |  |  |
| Low-risk | 74 | 141 | 0.862 | 0.865 (0.176-4.254) | 0.316 | 0.571 (0.214-1.522) | 0.771 | 1.176 (0.383-3.612) |
| High-risk | 54 | 39 | 0.757 | 1.158 (0.463-2.896) | 0.779 | 1.123 (0.502-2.510) | 0.964 | 0.982 (0.440-2.189) |
| All patients | 128 | 180 | 0.155 | 1.736 (0.793-3.803) | 0.473 | 1.244 (0.678-2.283) | 0.167 | 1.549 (0.817-2.935) |

Note: CC = Concurrent chemotherapy; IC = Induction chemotherapy; AC = adjuvant chemotherapy; LRFS = locoregional recurrence-free survival; DMFS = distant metastasis-free survival; OS = overall survival.
