# Supplementary material for: A Nomogram for the Determination of the Necessity of Concurrent Chemotherapy in Patients With Stage II–IVa Nasopharyngeal Carcinoma
Source: Front Oncol. 2021 Sep 6;11:640077. doi: 10.3389/fonc.2021.640077 (PMC8450530; doi:10.3389/fonc.2021.640077)
Supplement: Supplementary file 7 [file DataSheet_1.doc]

**Chemotherapy:**

The regimens of induction chemotherapy contained TPF (docetaxel 60 mg/m² IV on day 1, cisplatin, 75 mg/m² IV on day 1 or within 3 days, 5-FU 600 mg/m² IV on days 1 to 5); the GP (gemcitabine 1000mg/m2 IV on days 1 and 8, and cisplatin 25mg/m2 IV on days 1–3), which were repeated every 3 weeks. Concurrent chemoradiotherapy consisted of each of 2 regimes: Cisplatin 80 mg/m2 IV every 3 weeks; Cisplatin 30–40 mg/m2 IV weekly. For patients who received adjuvant chemotherapy, PF (cisplatin, 80 mg/m2 IV on day 1, 5-Fu 800 mg/m2/d continuously IV on day 1–5) or TPF regimen was repeated every 3 weeks.

**Radiotherapy:**

Gross tumor volume of nasopharynx (GTVnx) and gross tumor volume of cervical lymph node (GTVnd) were defined as visible tumour and the positive lymph nodes, respectively. Clinical target volume (CTV)-1 contained nasopharynx primary tumour with an additional a 5–10mm margin (2–3mm posteriorly adjacent to the spinal cord or brainstem). CTV-2 contained CTV-1 with the selective neck IB to V area and subclinical sites. The planning GTVnx/nd (PGTVnx/nd), the planning CTV-1 (PCTV-1) and the planning CTV-2 (PCTV-2) were obtained by expanding the corresponding GTVnx/nd, CTV-1 and CTV-2 with an expanded margin of 3 mm, respectively. PGTVnx/nd, PCTV-1 and PCTV-2 are prescribed to 69.96/73.92, 59.4, and 54 Gy, respectively, with 33-fraction (2.12 or 2.24 Gy per fraction) scheme for 6-7 weeks using 6-MV photons.

We used the Philips Pinnacle^3^ Planning System v.9.0 for IMRT planning. Direct machine parameter optimization (DMPO) module was adopted for the planning, which used 7–9 angles to evenly separate coplanar fields. The minimum segment area was set to 5 cm^2^, and minimum segment MU was 4-5 MUs. A collapsed-cone convolution-superposition algorithm was used to calculate dosage, with a dose grid resolution of 3 mm.
