# Supplementary material for: A Nomogram for the Determination of the Necessity of Concurrent Chemotherapy in Patients With Stage II–IVa Nasopharyngeal Carcinoma
Source: Front Oncol. 2021 Sep 6;11:640077. doi: 10.3389/fonc.2021.640077 (PMC8450530; doi:10.3389/fonc.2021.640077)
Supplement: Supplementary file 8 [file DataSheet_2.docx]

**IPTW code.：**

library(foreign)

library(RISCA)

library(survminer)

bc <- read.spss("Breast cancer survival agec.sav",

use.value.labels=F, to.data.frame=T)

bc <- na.omit(bc)

bc$CCgroup<-as.factor(bc$CCgroup)

names(bc)

fit=coxph(Surv(time,status) ~ CCgroup+Paper+Stage+Plan_0_GTVnd_Volume+T+N,data=bc)

summary(fit)

fit1 <- survfit(Surv(time,status) ~ CCgroup, data = bc)

plot(fit1, ylab="Confounder-adjusted survival",

xlab="Time post-transplantation (years)", col=c(1,2), grid.lty=1)

ggsurvplot(fit1, data = bc)

pr<- glm(CCgroup~ Paper+Stage+Plan_0_GTVnd_Volume+T+N, data=bc,

family=binomial(link = "logit"))

pr1<-pr$fitted.values

W <- (bc$CCgroup==1) * (1/pr1) + (bc$CCgroup==0) * (1)/(1-pr1)

fit.IPTW=coxph(Surv(time,status) ~ CCgroup+Paper+Stage+Plan_0_GTVnd_Volume+T+N,

data=bc,weights=W)

summary(fit.IPTW)

fit.ipw<-ipw.survival(times=bc$time, failures=bc$status,

variable=bc$CCgroup, weights=W)

plot(fit.ipw, ylab="Confounder-adjusted survival",

xlab="Time post-transplantation (years)", col=c(1,2), grid.lty=1)

fit2.ipw <- survfit(Surv(time,status) ~ CCgroup, data = bc,weights = W)

ggsurvplot(fit2.ipw, data = bc)

------------------------------------
